# Genetic polymorphisms in folate pathway enzymes, DRD4 and GSTM1 are related to temporomandibular disorder

**DOI:** 10.1186/1471-2350-12-75

**Published:** 2011-05-26

**Authors:** Angel Aneiros-Guerrero, Ana M Lendinez, Arturo R Palomares, Beatriz Perez-Nevot, Lidia Aguado, Alvaro Mayor-Olea, Maximiliano Ruiz-Galdon, Armando Reyes-Engel

**Affiliations:** 1 Department of Biochemistry and Molecular Biology, Faculty of Medicine, University of Málaga, Spain; 2 Hospital Clínico Universitario Virgen de la Victoria, Málaga, Spain

## Abstract

**Background:**

Temporomandibular disorder (TMD) is a multifactorial syndrome related to a critical period of human life. TMD has been associated with psychological dysfunctions, oxidative state and sexual dimorphism with coincidental occurrence along the pubertal development. In this work we study the association between TMD and genetic polymorphisms of folate metabolism, neurotransmission, oxidative and hormonal metabolism. Folate metabolism, which depends on genes variations and diet, is directly involved in genetic and epigenetic variations that can influence the changes of last growing period of development in human and the appearance of the TMD.

**Methods:**

A case-control study was designed to evaluate the impact of genetic polymorphisms above described on TMD. A total of 229 individuals (69% women) were included at the study; 86 were patients with TMD and 143 were healthy control subjects. Subjects underwent to a clinical examination following the guidelines by the Research Diagnostic Criteria for Temporomandibular Disorders (RDC/TMD). Genotyping of 20 Single Nucleotide Polymorphisms (SNPs), divided in two groups, was performed by multiplex minisequencing preceded by multiplex PCR. Other seven genetic polymorphisms different from SNPs (deletions, insertions, tandem repeat, null genotype) were achieved by a multiplex-PCR. A chi-square test was performed to determine the differences in genotype and allelic frequencies between TMD patients and healthy subjects. To estimate TMD risk, in those polymorphisms that shown significant differences, odds ratio (OR) with a 95% of confidence interval were calculated.

**Results:**

Six of the polymorphisms showed statistical associations with TMD. Four of them are related to enzymes of folates metabolism: Allele G of Serine Hydoxymethyltransferase 1 (SHMT1) rs1979277 (OR = 3.99; 95%CI 1.72, 9.25; p = 0.002), allele G of SHMT1 rs638416 (OR = 2.80; 95%CI 1.51, 5.21; p = 0.013), allele T of Methylentetrahydrofolate Dehydrogenase (MTHFD) rs2236225 (OR = 3.09; 95%CI 1.27, 7.50; p = 0.016) and allele A of Methionine Synthase Reductase (MTRR) rs1801394 (OR = 2.35; 95CI 1.10, 5.00; p = 0.037). An inflammatory oxidative stress enzyme, Gluthatione S-Tranferase Mu-1(GSTM1), null allele (OR = 2.21; 95%CI 1.24, 4.36; p = 0.030) and a neurotransmission receptor, Dopamine Receptor D4 (DRD4), long allele of 48 bp-repeat (OR = 3.62; 95%CI 0.76, 17.26; p = 0.161).

**Conclusions:**

Some genetic polymorphisms related to folates metabolism, inflammatory oxidative stress, and neurotransmission responses to pain, has been significantly associated to TMD syndrome

## Background

Temporomandibular joint disorder (TMD) is characterized by a set of symptoms related to the muscles and joints that reside between the mandibular condyle and the temporal bone. The most common symptoms are joint sounds, pain, and limited joint movement [1].

According to the NIDCR [2] (National Institute of Dental and Craniofacial Research), 5-12% of the population suffers or has suffered at some time from problems related to temporomandibular joint disorder (TMD) [3]. It is considered the most common cause of chronic pain in the orofacial region.

There are multiple factors that could cause or contribute to TMD disorder, including trauma, several types of arthritis, dental problems, autoimmune diseases, psychological and hormonal factors [4] and also commonly associated with other related symptoms in the head and neck [5].

Due to its etiological and symptomatic variety, it is assumed that a combination of factors related to environmental and/or nutritional stress could be an underlying cause of TMD [6-9]. As for genetic factors, studies of twins and familial segregation suggest that TMD is not a hereditary disease [10,11]. Nevertheless, individuals are not equally susceptible to TMD, and different genetic variants can increase the predisposition to a particular development of the disorder [12-22].

TMD is a problem that affects women two times more than men and its causes are not completely known. Certain hormones can increase inherent genetic vulnerability to TMD, which explains the larger predisposition of women of reproductive age [4,23]. The relationship between oestrogens and TMD has been studied previously [24,25]; specifically, the polymorphisms of the receptor gene ER-α, *Pvu *II (rs2234693) and *Xba *I (rs9340799), seem to be related to several forms of osteoarthritis, including TMD [17,19].

Folate metabolism can influences the final form of any growing tissue due not only to its participation in nucleic acid synthesis, but also to its known function in regulating DNA and protein methylation. Folate deficiency can cause central nervous system irritability, depression, weight loss, and anaemia. Nutritional deficiencies and, more specifically, abnormally low levels of vitamins B1, B6, B12, and/or folic acid are considered factors that perpetuate pain and myofascial dysfunction, and these deficiencies are frequent in cases of TMD mechanical stress [6].

Other mechanisms that have been related to TMD are factors related to psychopathological and pain perception, which can also be influenced by genetic variability. It has been described that the variants of a polymorphic region linked to gene SLC6A4 of the serotonin transporter (5-HTT gene-linked polymorphic region, 5-HTTLPR) [22] could be related to pain perception and temporomandibular disorder [18]. The 44 bp insertion/deletion polymorphism of the SLC6A4 gene has been extensively studied and frequently associated with the dopamine receptor gene DRD4; DRD4 variants have been associated with several clinical cases related to behavior, psychopathologies, and pain [17,26,27].

The following objective was to study the implication of 27 polymorphisms located on 17 genes and their relation with TMD. It has been designed under the hypothesis of that TMD is a multifactorial syndrome related to a critical period of human life that have a genetic and epigenetic basis both associated to folate metabolism, oxidative stress, hormones and neurotransmission.

## Methods

### Study design

A case-control study was designed to evaluate the impact of gene polymorphisms above described on TMD. A total of 229 individuals (69% women) were included at the study; 86 were TMD patients and 143 were healthy control subjects. The sample size was calculated using the prevalence of TMD and allele and genotypes frequencies of the polymorphism selected based on their known frequency in our population.

### Patient recruitment

Thirty five patients diagnosed with TMD who were admitted consecutively to private dental surgeries ('Clínica Rincon' and 'Clínica Dental Aneiros' in Malaga, Spain), during a period of 8 months were included in this study. In addition, 556 volunteer students from Malaga University (57% female and 43% male) were invited to participate in the study. They answered a questionnaire for TMD (Table 1) [28]. Ninety eight individuals responded affirmatively to four o more question of the questionnaire. So, they were invited to be clinically evaluated following the guidelines from The Research Diagnostic Criteria for Temporomandibular Disorders (RDC/TMD) [29] by a dentist who had experience in TMD evaluation. Fourteen subjects were excluded because they could not come to the physical examination and 33 had not clinical signs of TMD. Case group was 86 patients (35 previously diagnosed and 51 diagnosed volunteers), 64 women (74%), mean age 19.4 ± 2.9, and 22 men (26%) mean age 23.6 ± 4.1. Exclusion criteria for TMD patients were clinical history of traumatic injuries on head and neck, cranial anomalies of known or unknown etiology, inflammatory chronical disease component, mental retardation, drug dependence, and somatic or neurological illnesses.

**Table 1:** Questionnaire form for TMD

1. Do you have difficulty or pain, or both, when opening your mouth, as for instance, when yawning?
2. Does your jaw get stuck, locked, or go out?

3. Do you have difficulty or pain, or both, when chewing, talking, or using your jaws?

4. Are you aware of noises in the jaw joints?

5. Do you have pain in or about the ears, temples, or cheeks?

6. Does your bite feel uncomfortable or unusual?

7. Do you have frequent headaches?

8. Have you had a recent injury to your head, neck, or jaw?

9. Have you previously been treated for a jaw joint problem? If so, when?

From *J Am Dent Assoc 1990, 120: 253-254*

The control group was built from the volunteers who did not answer affirmatively to any question. All participants were healthy, without any signs or symptoms of TMD and age matched to the TMD group. In short, control group consisted in 143 subjects, 94 women (66%), mean age 19,6 ± 3.3, and 49 men (34%), mean age 22,3 ± 4.1.

All the participants signed an informed consent form, and their personal data were recorded by interviewers. The study protocol was approved by Ethics Committee of Malaga University and conformed to the Helsinki Declaration.

### Samples and DNA isolation method

After obtaining informed consent, oral mucosa samples were collected from the 229 subjects. DNA isolation was performed using the salting out method, as described by Martinez et al. [30], applied to the oral mucosa.

### Genotyping

Three platforms were designed to perform the genotyping. Two of them for 20 Single Nucleotide Polymorphisms (SNP1, SNP2) and the third one for other polymorphisms of 7 gene variants (deletions, insertions, tandem repeat, null genotype). Primers were designed using the primer analysis software *Oligo*, version 4.0 and synthesized commercially. The primers sequences are shown in table 2. To verify the validity and reliability of the techniques, the quality of each isolated polymorphism was checked in a simplex reaction.

**Table 2:** Information of the polymorphisms included in the study

Name	Reference	SNP/F	Location	A1	A2	Forward Primers	Reverse Primers	Minisequencing Primers	**Mult**.
**SHMT1**	rs1979276	SNP	3'-UTR	A	G	CTGGCAGGGGATAAGTACCAG	GTCAACAGTTCCCCTTTGGA	CCGGAGGACCCCCAC	SNP1
	rs1979277	SNP	EXON	A	G	CTGGCAGGGGATAAGTACCAG	GTCAACAGTTCCCCTTTGGA	(T)31-GCCAGGCAGAGGGAAGA	SNP1
	rs643333	SNP	5'-UTR	A	C	TGGACGCACATTTGTCCTAC	ATCAGAGAGCGCAGCCAAG	(T)24-ACCTGCAGAACTGACCC	SNP1
	rs638416	SNP	5'-UTR	C	G	TGGACGCACATTTGTCCTAC	ATCAGAGAGCGCAGCCAAG	(T)91-GCAGGGCCTGTTTCTCC	SNP1
	rs3783	SNP	3'-UTR	C	G	CTGGCAGGGGATAAGTACCAG	GTCAACAGTTCCCCTTTGGA	(T)115-GGGGTCCTCCGGCAG	SNP1
									
**TYMS**	rs2853542	SNP	5'-UTR	C	G	GTGCCACACCCGTGGCTCC	GCCACAGGCATGGCGCGG	(T)84-GGGACGGAGGCAGGC	SNP1
	rs34489327	F	3'-UTR	I	D	PET-CAAATCTGAGGGAGCTGAGT	CAGATAAGTGGCAGTACAGA		F
	rs34743033	F	5'-UTR	2R	3R	VIC-GTGCCACACCCGTGGCTCC	GCCACAGGCATGGCGCGG		F
									
**TCN**	rs9606756	SNP	EXON	A	G	GGAGAAGGCCCTGGTAACG	CTTCCTTGGTCCCAGCCTG	(T)64-GGCTGTCCATCTCTGGTA	SNP1
	rs1801198	SNP	EXON	C	G	GGGAAAGAGACCCTGGAGC	GCTGGGAAATCATGAGAGC	(T)58-CCCAGTTCTGCCCCA	SNP2
									
**MTHFR**	rs1801131	SNP	EXON	A	C	CTTTGGGGAGCTGAAGGACTACTAC	CACTTTGTGACCATTCCGGTTTG	(T)71-AGGAGCTGACCAGTGAAG	SNP2
	rs1801133	SNP	EXON	C	T	GTGGTCTCTTCATCCCTCG	GACGGTGCGGTGAGAGTG	GAAGGTGTCTGCGGGAG	SNP2
									
**CBS**	rs5742905	SNP	EXON	C	T	GGTTCTTGGGTTTCTCATCC	CTCCGTCTGGTTCAGCTCC	(T)42-GCGCCCTCTGCAGATCA	SNP2
	cbs844ins68	F	INTR/EXON	S	L	6FAM-GTTGTTAACGGCGGTATTGG	GTTGTCTGCTCCGTCTGGTT		F
									
**ABCB1**	rs1045642	SNP	EXON	C	T	GCTGAGAACATTGCCTATGG	TAAGGGTGTGATTTGGTTGC	(T)34-GGTGTCACAGGAAGAGAT	SNP2
**ATIC**	rs2372536	SNP	EXON	C	G	CCTAGATAGCTGTAAACCAC	GTAATCCCAAAACACAATC	(T)18-CCACAGCCTCCTCAACA	SNP1
**BHMT**	rs3733890	SNP	EXON	A	G	TGTGAACTGCCACTTTGACC	ATGGGAATTCTGGGAGATCG	ATCAGGTGAGCTTTCAGT	SNP1
**DHFR**	DHFR19bpdel	F	INTRON	+	-	ACGGTCGGGGTGGCCGACTC	6FAM-AAAAGGGGAATCCAGTCGG		F
**GSTM1**	GSTM1del	F	EXON	+	-	PET-GAACTCCCTGAAAAGCTAAAGC	GTTGGGCTCAAATATACGGTGG		F
									
**MTHFD1**	rs2236225	SNP	EXON	C	T	CCCACTTTGAAGCAGGATTG	CATCCCAATTCCCCTGATG	(T)59-AACAAGCTTGAGTGCGATC	SNP1
**MTR**	rs12749581	SNP	EXON	A	G	GCATTGACCATTACTACACC	TCCAAAGCCTTTTACACTCC	AATATGAAGATATTAGACAGG	SNP2
**MTRR**	rs1801394	SNP	EXON	A	G	TTTCAGTTTCACTGTTACATGC	GTAACGGCTCTAACCTTATCG	(T)51-ACCACAGCTTGCTCACA	SNP1
**RFC1**	rs1051266	SNP	EXON	A	G	TTCCAGGCACAGTGTCACC	CCGCGTGAAGTTCTTGTCG	(T)40-CCGGTCCTGGCGGC	SNP1
									

**ESR1**	rs9340799	SNP	INTRON	A	G	AGGGTTATGTGGCAATGACG	CTGCACCAGAATATGTTACC	(T)_4_-AGACCCTGAGTGTGGTCT	SNP2
	rs2234693	SNP	INTRON	C	T	AGGGTTATGTGGCAATGACG	CTGCACCAGAATATGTTACC	(T)_28_-AGTTCCAAATGTCCCAGC	SNP2

**DRD4**	drd4-48bptr	F	EXON	S	L	NED-GCTGCTGCTCTACTGGGC	CCCGGCCGGTGATCTTGG		F
**SLC6A4**	SCL6A45-HTTLPR	F	5-HTTLPR	S	L	VIC-GCGTTGCCGCTCTGAAGTC	GTGCCACCTAGACGCCAGG		F

SNP = Single Nucleotide Polimorphism; F = Fragment or Repeat; A1 = Allele 1; Allele2; MULT = Multiplex Reaction;SHMT1 = Serine Hydroxymethyltransferase1; TYMS = Thymidylate Synthetase; TCN = Transcobalamin II; MTHFR = 5,10-Methylenetetrahydrofolate Reductase; CBS = Cystathionine Beta-Synthase; ABCB1 = ATP-Binding Cassette B1; ATIC = 5-Aminoimidazole-4-Carboxamide Ribonucleotide Formyltransferase/IMP Cyclohydrolase; BHMT = Betaine-Homocysteine Methyltransferase; DHFR = Dihydrofolate Reductase; MTHFD1 = Methylenetetrahydrofolate Dehydrogenase 1; MTR = 5-Methyltetrahydrofolate-Homocysteine S-Methyltransferase; MTRR = Methionine Synthase Reductase; RFC1 = Reduced Folate Carrier 1; ESR1 = Estrogen Receptor 1; DRD4 = Dopamine Receptor D4; SLC6A4 = Solute Carrier Family 6

### SNPs

Genotyping of SNPs was performed by multiplex minisequencing preceded by multiplex PCR [31]. This technique consists of three steps:

1) Amplification of regions flanking the SNPs by multiplex PCR; PCR reactions were performed in a volume of 10 μL with 100 ng of genomic DNA, 1×Amplitaq Gold ^® ^Buffer (Applied Biosystems), MgCl_2 _1,5 mM, dNTPs 0,2 mM, 1 unit of Amplitaq Gold^® ^DNA Polymerase (Applied Biosystems) and Primer Mix (primer concentrations ranged between 0,1-0,6 μM). Amplification was achieved in a 2720 Thermal Cycler^®^(Applied Biosystems) and consisted of 94°C for 5 minutes, followed by 35 cycles for 30 s at 94°C, 30 s at 58°C; 30 s at 72°C and a final extension for 7 min at 72°C. PCR products were checked in a 2% agarose gel, stained with SYBR^® ^Safe DNA gel stain (Invitrogen) which resulted in 100 to 400 bp sized bands. In order to eliminate the excess of primers and dNTPs, the PCR products were digested by an 'ExoCiAP' mix consisting of 2 units/10 μL PCR *E. coli *exonuclease I (Exo I, Takara^®^) and 5 units/10 μLPCR alkaline phosphatase (CiAP, Takara^®^) incubated at 37°C for 60 minutes. The enzymes were afterwards inactivated by heating at 80°C for 20 minutes

2) Multiplex mini-sequencing for each locus of the SNP multiplexes (SNP1, SNP2), a mini-sequencing primer with the 3'-end adjacent to the target SNP was designed (see Table 2) to anneal with the PCR product. A mini-sequencing reaction extends this primer, producing different products for each allele. We performed the reaction in a 11 μL volume with 4 μL purified PCR product and 6 μL of the mini-sequencing primers mix (2 pM/μL) and 1 μL of SNaPshot^® ^multiplex kit (Applied Biosystems). The mini-sequencing conditions consisted of 40 cycles at 96°C-10 s; 50°C-7 s; 60°C-30 s. After that, we proceeded to purify the sample using 1 unit of *alkaline phosphatase *(Takara^®^) at 37°C for 1 hour and then at 80°C for 20 min.

3) Analysis of mini-sequencing products by capillary electrophoresis: We mixed 4 μl of the purified mini-sequencing products with 10 μl of HiDi™ formamide and 0.2 *μ*l of GeneScan-120 LIZ size standard (Applied Biosystems) and denatured at 95°C for 5 minutes. The fluorescently labeled products were resolved by capillary electrophoresis on an ABI PRISM 3130 Genetic Analyzer (Applied Biosystems). The DS02 matrix (Applied Biosystems) was used at full speed for 17 minutes with 8 seconds of injection. The resulting data were analyzed with GeneMapper™ 4.0 Software (Applied Biosystems).

### Other polymorphisms

To detect other polymorphisms different from SNPs (deletions, insertions, tandem repeat, null genotype), a multiplex PCR assay was performed under the following concentrations (in a volume of 10 μL): 100 ng of genomic DNA, 1× GoTaq^® ^FlexiBuffer (Promega), MgCl_2 _1,5 mM, dNTPs 0,2 mM, Primer Mix (primer concentrations ranged between 0,1-0,6 μM), Betaine 50 mM and 1 unit of Go Taq^® ^Flexi DNA Polymerase (Promega). The labeled primers are shown in table 2. Amplification was achieved in a 2720 Thermal Cycler^® ^(Applied Biosystems) and consisted of 94°C for 5 minutes, followed by 35 cycles for 30 s at 94°C, 45 s at 58°C; 1 min at 72°C and a final extension for 7 min at 72°C. We mixed 4 μl of PCR products with 10 μl of HiDi™ formamide and 0.2 *μL *of GeneScan-600 LIZ size standard (Applied Biosystems) and denatured at 95°C for 5 minutes. The fluorescently labeled products were resolved by capillary electrophoresis on an ABI PRISM 3130 Genetic Analyzer (Applied Biosystems). The DS33 matrix (Applied Biosystems) was used at full speed for 45 minutes with 5 seconds of injection. The sizes of the alleles were analyzed with GeneMapper Ver. 4.0 (Applied Biosystems) software.

### Statistics

All analyses were performed using Statistical Package for Social Sciences statistical software (SPSS v. 16.0 for Macintosh; SPSS Inc., Chicago, IL). To determine the differences in genotype and allelic frequency of each studied polymorphism between TMD patients and healthy subjects, a chi-square test was used. Any p value < 0.05 was considered as a statistical difference. In those polymorphisms that shown significant difference in genotype, the risk between subjects with TMD and healthy controls was evaluated by Cochran's-Haenzel odds ratios with a 95% confidence interval using sex as confounding factor.

## Results

Genotyping was performed for the 229 individuals. The results are summarized in Table 3. Not all of the samples were valid for all genotypes, because of that, the number of individuals can vary from one polymorphism to another.

**Table 3:** Allele and genotypes frequencies in TMD and control groups

GENE	REFERENCE	ALELLE	PATIENTS				CONTROLS					ODDS RATIOS			
			**G1**	**G2**	**G3**	**A1/A2**	**G1**	**G2**	**G3**	**A1/A2**	**Χ^2^genotype**	**Allele**	**OD**	**95%CI**	**p**

**SHMT1**	rs1979276	A/G	7 (0,10)	36 (0,49)	30 (0,41)	0,34/0,66	24 (0,20)	54 (0,45)	41 (0,34)	0,43/0,57					
	rs1979277	A/G	8 (0,11)	31 (0,44)	32 (0,45)	0,33/0,67	35 (0,34)	28 (0,27)	41 (0,39)	0,47/0,53	0,002	G	3,99	1,72-9,25	0,002
	rs643333	A/C	3 (0,04)	32 (0,43)	39 (0,53)	0,26/0,74	10 (0,08)	47 (0,39)	64 (0,53)	0,28/0,72	ns		-		
	rs638416	C/G	19 (0,24)	39 (0,50)	20 (0,26)	0,49/0,51	12 (0,10)	50 (0,41)	60 (0,49)	0,30/0,70	0,001	C	2,80	1,51-5,21	0,013
	rs3783	C/G	1 (0,01)	27 (0,36)	46 (0,62)	0,20/0,80	2 (0,02)	33 (0,28)	85 (0,71)	0,15/0,85	ns		-		
															
**TYMS**	rs2853542	C/G	55 (0,73)	20 (0,27)	0 (0,00)	0,87/0,13	96 (0,76)	30 (0,24)	0 (0,00)	0,88/0,12	ns		-		
	rs34489327	DI	9 (0,13)	25 (0,35)	38 (0,53)	0,30/0,70	7 (0,08)	32 (0,34)	54 (0,58)	0,25/0,75	ns		-		
	rs34743033	2R3R	12 (0,17)	36 (0,50)	24 (0,33)	0,42/0,58	10 (0,12)	55 (0,64)	21 (0,24)	0,44/0,56	ns		-		
															
**TCN**	rs9606756	A/G	57 (0,75)	16 (0,21)	3 (0,04)	0,86/0,14	85 (0,73)	30 (0,26)	1 (0,01)	0,86/0,14	ns		-		
	rs1801198	C/G	30 (0,38)	38 (0,49)	10 (0,13)	0,63/0,37	56 (0,46)	61 (0,50)	6 (0,05)	0,70/0,30	ns		-		
															
**MTHFR**	rs1801131	A/C	44 (0,57)	25 (0,32)	8 (0,10)	0,73/0,27	64 (0,53)	47 (0,39)	10 (0,08)	0,72/0,28	ns		-		
	rs1801133	C/T	27 (0,35)	37 (0,48)	13 (0,17)	0,59/0,41	43 (0,34)	62 (0,49)	22 (0,17)	0,58/0,42	ns		-		
															
**CBS**	rs5742905	C/T	0 (0,00)	12 (0,18)	54 (0,82)	0,09/0,91	2 (0,02)	27 (0,25)	81 (0,74)	0,14/0,86	ns		-		
	cbs844ins68	SL	59 (0,84)	11 (0,16)	0 (0,00)	0,92/0,08	68 (0,81)	14 (0,17)	2 (0,02)	0,89/0,11	ns		-		
															
**ABCB1**	rs1045642	C/T	28 (0,36)	38 (0,49)	12 (0,15)	0,60/0,40	30 (0,24)	64 (0,51)	31 (0,25)	0,50/0,50	ns		-		
**ATIC**	rs2372536	C/G	23 (0,49)	18 (0,38)	6 (0,13)	0,68/0,32	44 (0,43)	42 (0,41)	17 (0,17)	0,63/0,37	ns		-		
**BHMT**	rs3733890	A/G	11 (0,15)	29 (0,40)	32 (0,44)	0,35/0,65	17 (0,14)	54 (0,45)	49 (0,41)	0,37/0,63	ns		-		
**DHFR**	DHFR19del	-+	22 (0,31)	31 (0,43)	19 (0,26)	0,52/0,48	29 (0,31)	48 (0,52)	16 (0,17)	0,57/0,43	ns		-		
**GSTM1**	GSTM1del	-	17 (0,23)	56 (0,77)		0,62/0,38	39 (0,40)	58 (0,60)		0,70/0,30	0,015	null	2,21	1,24-4,36	0,030
**MTHFD1**	rs2236225	C/T	7 (0,09)	47 (0,63)	21 (0,28)	0,41/0,59	28 (0,24)	70 (0,60)	18 (0,16)	0,54/0,46	0,011	T	3,09	1,27-7,50	0,016
**MTR**	rs1805087	A/G	55 (0,71)	20 (0,26)	2 (0,03)	0,84/0,16	80 (0,63)	42 (0,33)	4 (0,03)	0,80/0,20	ns		-		
**MTRR**	rs1801394	A/G	28 (0,37)	37 (0,49)	11 (0,14)	0,61/0,39	29 (0,25)	54 (0,47)	33 (0,28)	0,48/0,52	0,046	A	2,35	1,10-5,00	
**RFC1**	rs1051266	A/G	15 (0,20)	42 (0,55)	19 (0,25)	0,47/0,53	28 (0,23)	59 (0,49)	33 (0,28)	0,48/0,52	ns		-		

**ESR1**	rs9340799	A/G	37 (0,48)	35 (0,45)	5 (0,06)	0,71	55 (0,43)	59 (0,46)	13 (0,10)	0,67/0,33	ns		-		
	rs2234693	C/T	13 (0,17)	44 (0,56)	21 (0,27)	0,45	32 (0,25)	60 (0,48)	34 (0,27)	0,49/0,51	ns		-		

**DRD4**	drd448bptr	SL	50 (0,96)	2 (0,04)	0 (0,00)	0,98	69 (0,87)	7 (0,09)	3 (0,04)	0,92	0,039	L	3,12	0,76-17,2	0,161

**SCL6A4**	5-HTTLPR	SL	20 (0,43)	16 (0,35)	10 (0,22)	0,61	31 (0,40)	32 (0,41)	15 (0,19)	0,60	ns		-		

ns = no statistical difference

### Serine Hidroximetil Transferase 1 gene (SHMT1)

**Figure 1:**
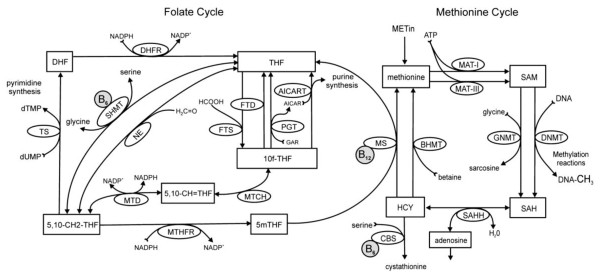
**The Folate and methionine cycles**. Abreviations: 10f-THF: 10-formyltetrahydrofolate; 5,10-CH2-THF: 5,10-methylenetetrahydrofolate; 5,10-CH-THF: 5,10-methenyltetrahydrofolate; 5mTHF: 5-methyltetrahydrofolate; AICART: Aminoimidazolecarboxamide ribotide transformylase; ATIC: 5-aminoimidazole-4-carboxamide ribonucleotide formyltransferase/imp cyclohydrolase; BHMT: Betaine-homocysteine methyltransferase; CBS: Cystathionine beta-synthase; DHF: Dihydrofolate reductase; DNMT: S-adenosylmethionine-dependent methyltransferases; DNMT, DNA methyltransferase; FTD: 10-formyltetrahydrofolate dehydrogenase; FTS: 10-formyltetrahydrofolate synthase; GNMT: glycine N-methyltransferase; GST: Glutathione S-transferase; MAT: methionine adenosyltransferase; MTCH: 5,10-methylenetetrahydrofolate cyclohydrolase; MTD: Methylenetetrahydrofolate dehydrogenase; MTHFR: 5,10-methylenetetrahydrofolate reductase; MTR: 5-methyltetrahydrofolate-homocysteine S-methyltransferase; MTRR: Methionine synthase reductase; NE: nonenzymatic interconversion of THF; PGT: phosphoribosyl glycinamidetransformylase; SAH: S-adenosylhomocysteine; SAHH: S-adenosyl homocysteine hydrolase; SAM: S-adenosylmethionine; SHMT: Serine hydroxymethyltransferase; TCN2: Transcobalamin II; THF: tetrahydrofolate; TS: thymidylate synthase; TYMS: Thymidylate synthetase.

This enzyme catalyzes the reversible conversion of serine to glycine, transferring methyl groups to tetrahydrofolate (THF), which is transformed into 5,10-methylenetetrahydrofolate (5,10-CH2-THF). During cellular proliferation, this is the predominant pathway for pyrimidine synthesis (see Figure 1).

From the four polymorphisms studied on the cytoplasmic SHMT1 in TMD patients, a significant increase was observed of the G allele of the polymorphism rs1979277 (Leu435Phe) when compared with controls (OR = 3.99; 95% CI 1.72, 9.25; p = 0,002). Regarding the polymorphism SHMT1 rs638416 of the promoter region (5'-UTR), a very significant increase was observed of the C allele and the CC genotype in TMD patients (OR = 2.80; 95% CI 1.51, 5.21; p = 0,013). Both polymorphisms are not linked [32], so their frequencies can be measured independently as a risk factor for TMD.

### Methylenetetrahydrofolate dehydrogenase 1 gene (MTHFD1)

The MTHFD1 gene encodes a protein that has three distinct enzyme activities: 5,10-methylenetetrahydrofolate dehydrogenase, 5,10-methylenetetrahydrofolate cyclohydrolase, and 10-formyltetrahydrofolate-synthetase. Each of these activities catalyzes one of three interconversion reactions of one-carbon derivatives of THF, which are substrates for the synthesis of methionine, thymidylate and purines. The trifunctional enzymatic activity is conferred by two larger domains: a terminal amino portion with dehydrogenase and cyclohydrolase activities and a larger domain with synthetase activity.

The transition from G to A (rs2236225) in the MTHFD1 gene that results in an alteration of ARG-653-GLN is clinically associated with susceptibility to neural tube defects (NTDs) sensitive to folates [33]. In our study, we found statistical differences in the alleles and genotypes frequencies between TMD patients and healthy controls. The T allele was more frequent in TMD patients (OR = 3.09; 95% CI 1.27, 7.50; p = 0,016).

### Glutathione S-transferase mu 1 (GSTM1) gene

This gene encodes mu-class glutathione S-transferase. Its function is the detoxification of electrophilic compounds, including carcinogens, drugs, toxins, and products of oxidative stress, by the conjugation with glutathione.

The GSTM1-null polymorphism is a deletion of the GSTM1 gene. The null variant of this gene has been associated with an increase of cancer and male infertility [34,35] possibly due to greater susceptibility to environmental toxins and carcinogens as well as an alteration in the toxicity and efficiency of certain substances.

Our study found a statistical increase (OR = 2.21; 95% CI 1.24, 4.36; p = 0.030) of the null variant in TMD patients compared to the control group.

### Methionine Synthase Reductase (MTRR) gene

MTRR is responsible for the regeneration of Methionine Synthase (MTR) by reductive methylation using SAM as a methyl donor.

The substitution of A by G (rs1801394) in the encoding region is translated in Ile22Met. The G allele produces an enzyme with less affinity for the substrate [36]. A statistical increase of the A allele (OR = 2.35; 95% CI 1.10, 5.00; p = 0.037) was observed in the TMD group.

### D4 receptor of Dopamine (DRD4) gene

DRD4-48bptr is the 48-bp VNTR in exon 3 of the DRD4, which encodes the third intracellular receptor loop and presents between 2 to 11 repeats. According to the number of repetitions, we can characterize the polymorphism as the short allele S-DRD4 (2 to 5 repetitions) and the long allele L-DRD4 (6 or more repeats) [37]. It was observed statistical differences on genotype frequencies in both groups (p = 0,039), but no statistical increase (OR = 3.12; 95% IC 0.76, 17.26, p = 0.181) of the S-DRD4 allele was found in patient group. The LL genotype was not found in any of the individuals with TMD.

## Discussion

Temporomandibular disorder has been associated with psychological dysfunctions [38], and sexual dimorphism [24]. Its occurrence also appears to coincide with puberty [25]. Currently, controversy exists as to the importance of underlying genetic and environmental nutritional factors, such as vitamin intake [7]. The folate-methionine axis takes part in both factors. In this axis, there are several polymorphic variants of the enzymes involved that together with folate dietary intake determines the metabolism of the axis. Furthermore, the transfer of methyl groups to DNA and proteins is one of the limiting factors for the correct development of the proliferating tissues. Therefore the latter is key for the continuous tissue growth and epigenetic modifications.

This work studied the implication of 27 polymorphisms located on 17 genes and their relation with TMD. It has been designed under the hypothesis of that TMD is a multifactorial syndrome related to a critical period of human life that have a genetic and epigenetic basis [12-22]. The study of genes related to folate cycle is directly involved in the two ways: the genetic, by gene polymorphisms, and epigenetic by the nutritional habits because of the folate intake. Deficit of folate intake is known as the most prevalent vitamin deficit in human. The majority of the genes studied were related to folate metabolism even though there were others related to oxidative metabolism and hormonal and neurotransmission receptors. Of the17 genes studied of the folate cycle, 3 of them (SHMT, MTHFD, MTRR) showed significant associations with TMD. Significant changes in the allele and genotype frequencies between the TMD patients and controls were found.

SHMT catalyzes a reversible step of THF to 5,10-CH_2_-THF, which is a key substrate to obtain 5-methyl-THF and synthesize thymidylate. On the other hand, MTHFD1 provides the 5, 10CH_2_-THF substrate in addition to having a central role in the cycle due to its triple functionality. The 5-methyl-THF to methionine step is catalyzed by MTR in cooperation with TCN and MTRR, which are essential for the participation of vitamin B12 as a cofactor and the reactivation of MTR, respectively.

In our case, a higher significant frequency of the allele SHMT-rs1979277G was observed in TMD patients (0.67 vs 0.53). The Allele G has been related to higher levels of folates and homocysteine [39], this could mean that the enzyme activity is deviated to the THF and glycine synthesis versus to the serine and 5,10CH_3_THF substrate of the MTHFR which provide 5'CH_3_THF needed to the methionine synthesis by mean of homocysteine. The increase of homocysteine, classified as an oxidant, could favour inflamatory process. However the effect of this polymorphism on enzyme activity is still controverted [39].

Statistical differences were also observed in allelic frequency of the C allele the SNP rs638416, in the promoter region of the SHMT gene. SHMT not only provides one-carbon units for thymidylate biosynthesis but also generates a pool of methylenetetrahydrofolate for SAM synthesis by means of serine synthesis. In addition, the SHMT gene has the capacity to sequester 5-methyl-THF and therefore inhibit the synthesis of methionine and SAM, which is the methyl donor for DNA and proteins [39]. Therefore, greater SHMT activity would produce higher 5-methyl-THF sequestration, which would reduce SAM synthesis. Cellular deficits of SAM could induce lesser methylation on the novo synthetized DNA on inflamatory tissues, which can take to higher gene expresión levels, generating a positive feedback on inflammation and pain.

TMD patients and healthy controls had very significantly different genotypic frequencies of the SNP rs2236225 of the MTHFD gene. The triple functionality of the MTHFD enzyme and the central role it plays in the folate cycle make it one of the key points for the balance of this metabolic route [24]. Therefore, it is not difficult to believe that any genetic alteration that affects its expression or activity would have consequences for methyl transfer and consequently on epigenesis.

The results of the present work show that the polymorphic variants SHMT-rs1979277, rs638416, and MTHFD1-rs2236225 found in the folate cycle were significantly associated with TMD. They coincided with a global decrease in the availability of methyl groups via a decrease of the substrate 5,10-CH_2_-THF (SHMT and MTHFD1), while the SNP MTRR-rs1801394 did not follow the same pattern. The wild allele A, predominant in TMD, which means greater efficiency in the pathway of homocysteine to methionine, has the same final effect of the variants of SHMT and MTHFD, an increase of THF levels.

TMD have been associated to oxidative stress of the temporomandibular joint [40-44]. Regarding GTSM1, we found a significant number of individuals with TMD (OD = 2.21; 95%CI 1.24, 4.35; p = 0.030) with the null variant of the gene. It has been published that oxidative stress, which leads to the production of NO and peroxynitrite, is harmful to DNA in response to the excessive mechanical overload, which in turn promotes synovial hyperplasia in the temporomandibular joint [40]. This suggests that a reduced detoxification capacity would make us more susceptible to TMD.

Previous studies on associations of TMD with alpha ESR1 polymorphisms have shown different results. One of them find a predisposition to TMD associated to ESR1 polymorphism in women [15] others find a relation between pain [17] or craniofacial morphology [19] in TMD women with ESR1 polymorphism, however other recent report does not find this association[12]. In the present study there were no statistical differences in their individualized allelic and genotypic frequencies or in the distinct haplotype combinations. However, a study on the association between these polymorphisms and the predisposition to TMD has recently been published and deepens the characterization of the diplotypes using novel mathematic algorithms[15], which we will take into account in future studies.

In contrast to others authors [18,21], significant variations were not found in the frequencies of the 5-HTTLPR (serotonin-transporter-linked promoter region) polymorphism of the serotonin receptor gene SCL6A4. However, we found a significant variation in the 48-bp VNTR polymorphism of the DRD4 gene. This polymorphism has been associated with pain processes, such as fibromyalgia [35] and migraine [36].

## Conclusions

In this study we have found new associations of polymorphic genes, related to folates, SHMT, MTHFD and MTR, oxidative stress GSTM1 and neurotransmission DRD4, with TMD. Concluding that the emergence of TMD in the tissues involved in joint morphogenesis in the final phase of maturity may occur due to multiple factors. Among those factors, we have found genetic polymorphisms involving folate deficiency, psycho-physical stress and the oxidative metabolism in cellular proliferation during puberty. The novelty of the results obtained in this study should be confirmed and extended in subsequent work with larger populations.

## Competing interests

The authors declare that they have no competing interests.

## Authors' contributions

AAG contributed to the acquisition, analysis and interpretation of data, and performed the drafting of the manuscript. AML and BPN carried out the molecular genetic analysis, interpretation of data and drafting the manuscript. ARP participated in the sequence alignment, design of the PCR multiplex's and drafted the manuscript. AMO performed statistical analysis and drafted the manuscript. LA contributed to the acquisition and interpretation of data. MRG participated in the design and coordination of the study and he helped to draft the manuscript. ARE conceived and designed the study, he oversaw the analysis, contributed to the interpretation of the results and preparation of the final manuscript. All authors read and approved the final manuscript

## Pre-publication history

The pre-publication history for this paper can be accessed here:

http://www.biomedcentral.com/1471-2350/12/75/prepub

## References

[B1] The TMJ association http://www.tmj.org

[B2] National Institute of Dental and Craniofacial Research http://www.nidcr.nih.gov

[B3] National Institute of Dental and Craniofacial Research http://www.nidcr.nih.gov/DataStatistics/FindDataByTopic/FacialPain

[B4] Wang J Chao Y Wan Q Zhu Z The possible role of estrogen in the incidence of temporomandibular disorders Med Hypotheses 2008 71 564 567 10.1016/j.mehy.2008.05.011 18597950

[B5] Armijo Olivo S Magee DJ Parfitt M Major P Thie NM The asssociation between the cervical spine, the stomatognathic system and craniofacial pain: a critical review J Orofac Pain 2006 20 271 287 17190026

[B6] Mehra P Wolford LM Serum nutrient deficiencies in the patient with complex temporomandibular joint problems Proc (Bayl Univ Med Cent) 2008 21 243 247 10.1080/08998280.2008.11928403 PMC2446412 18628971

[B7] Mäder R Deutsch H Siebert GK Gerbershagen HU Grühn E Behl M Kübler W Vitamin status of in patients with chronic cephalgia and dysfunction pain syndrome and effects of a vitamin supplementation Int J Vitam Nutr Res 1988 58 436 441 3072307

[B8] Johanson AK Johanson A Unell L Norring C Carlsson GE Eating disorders and sign and symptoms of temporomandibular disorders: a matched case-control study Sweed Dent J 2010 34 139 147 21121413

[B9] Slade GD Diatchenko L Ohrbach R Maixner W Orthodontic treatment, genetic factors and risk of temporomandibular disorder Sem Orthod 2008 14 146 156 10.1053/j.sodo.2008.02.005 PMC2486446 18663384

[B10] Raphael KG Marbach JJ Gallagher RM Dohrenwend BP Myofascial TMD does not run in families Pain 1999 80 15 22 10.1016/S0304-3959(98)00180-8 10204713

[B11] Heiberg A Heloe B Heiberg AN Heloe LA Magnus P Berg K Nance WE Myofascial pain dysfunction (MPD) syndrome in twins Community Dent Oral Epidemiol 1980 8 434 6 10.1111/j.1600-0528.1980.tb01323.x 6942960

[B12] Kim BS Kim YK Yun PY Lee E Bae J The effects of estrogen receptor polymorphism on the prevalence of symptomatic temporomandibular disorders J Oral Maxillofac Sur 2010 68 2975 2979 10.1016/j.joms.2010.02.023 20656393

[B13] Nackley AG Diatchenko L Assesing potential functionality of catechol-O-methyltransferase polymorphisms associated with pain and temporomandibular joint disorders Methods Mol Biol 2010 617 375 393 10.1007/978-1-60327-323-7_28 20336436

[B14] Tchivileva IE Lim PF Smith SB Slade GD Diatchenko L McLean SA Maixner W Effect of cathecol-O-methyltranferase polymorphism on response to propanolol therapy in chronic musculoskeletal pain: a randomized, doubled-blind, placebo-controlled, crossover pilot study Pharmacogenet Genomics 2010 20 239 248 20216107 10.1097/FPC.0b013e328337f9ab PMC2876724

[B15] Ribeiro-Dasilva MC Peres Line SR Leme Godoy dos Santos MC Arthuri MT Hou W Fillingim RB Rizzatti Barbosa CM Estrogen receptor-alpha polymorphism and predisposition to TNJ disorder J Pain 2009 10 527 533 10.1016/j.jpain.2008.11.012 19411060 PMC2749669

[B16] Etoz OA Ataoglu H Erdal ME Association between tryptophan hidroxylase gen polymorphism and painful non-osseus temporomandibular disorder Saudi Med J 2008 29 1352 1354 18813430

[B17] Kang SC Lee DG Choi JH Kim ST Kim YK Ahn AH Association between estrogen receptor polymorphism and pain susceptibility in female temporomandibular joint osteoarthritis patients Int J Oral Maxillofac Surg 2007 36 391 394 10.1016/j.ijom.2006.12.004 17391927

[B18] Ojima K Narita N Narita M Temporomandibular disorder is associated with serotonin transporter gene polymorphism in the Japanese population Biopsichosoc Med 2007 1 3 10.1186/1751-0759-1-3 PMC1805776 17371573

[B19] Lee DG Kim TW Kang SC Kim ST Estrogen receptor gene polymorphism and craniofacial morphology in female TMJ osteoarthritis patients Int J Oral Maxillofac Surg 2006 35 165 169 10.1016/j.ijom.2005.06.009 16154319

[B20] Mutlu N Erdal ME Herken H Oz G Bayazit YA T102C polymorphism of the 5-HT2A receptor gene may be associated with temporomandibular dysfunction Oral Dis 2004 10 349 352 10.1111/j.1601-0825.2004.01037.x 15533210

[B21] Herken H Erdal E Mutlu N Barlas O Cataloluk O Oz F Güray E Possible association of temporomandibular joint pain and dysfunction with a polymorphism in the serotonin transporter gene Am J Orthod Dentofacial Orthop 2001 120 308 313 10.1067/mod.2001.115307 11552131

[B22] Heils A Teufel A Petri S Stöber G Riederer P Bengel D Lesch KP Allelic variation of human serotonin transporter gene expression J Neurochem 1996 66 2621 2624 8632190 10.1046/j.1471-4159.1996.66062621.x

[B23] Landi N Manfredini D Lombardi I Casarosa E Bosco M 17-Beta-estradiol and progesterone serum levels in temporomandibular disorder patients Minerva Stomatol 2004 53 651 60 15894940

[B24] Halpern LR Levine M Dodson TB Sexual dimorphism and temporomandibular disorders (TMD) Oral Maxillofac Surg Clin North Am 2007 19 267 77 10.1016/j.coms.2007.01.012 18088884

[B25] Warren MP Fried JL Temporomandibular disorders and hormones in women Cells Tissue Organ 2001 169 187 192 10.1159/000047881 11455113

[B26] Benjamin J Osher Y Kotler M Gritsenko I Nemanov L Belmaker RH Ebstein RP Association between tridimensional personality questionnaire (TPQ) traits and three functional polymorphisms: dopamine receptor D4 (DRD4), serotonin transporter promoter region (5-HTTLPR) and catechol O-methyltransferase (COMT) Mol Psychiatry 2000 5 96 100 10.1038/sj.mp.4000640 10673775

[B27] Treister R Pud D Ebstein RP Laiba E Gershon E Haddad M Eisenberg E Associations between polymorphisms in dopamine neurotransmitter pathway genes and pain response in healthy humans Pain 2009 147 187 93 10.1016/j.pain.2009.09.001 19796878

[B28] McNeill C Mohl N Rugh J Tanaka T Temporomandibular disorders: diagnosis management, education, and research J Am Dent Assoc 1990 120 253 254 2179355 10.14219/jada.archive.1990.0049

[B29] Dworkin SF LeResche L Research diagnostic criteria for temporomandibular disorders: review, criteria, examinations and specifications, critique J Craniomandib Disord 1992 6 301 55 1298767

[B30] Martínez G Shaw EM Carrillo M Zanuy S A protein salting-out method applied in genomic DNA isolation from fish whole blood Biotechniques 1998 24 238 239 9494722 10.2144/98242bm14

[B31] Caravalho CM Pena SD Optimization of a multiplex minisequencing protocol for population studies and medical genetics Genet Mol Res 2005 4 115 125 16110434

[B32] Wang L Lu J An J Shi Q Spitz MR Wei Q Polymorphisms of cytosolic serine hydroxymethyltransferase and risk of lung cancer: a case-control analysis Lung Cancer 2007 57 143 51 10.1016/j.lungcan.2007.03.002 17420066 PMC2693017

[B33] De Marco P Merello E Calevo MG Mascelli S Raso A Cama A Capra V Evaluation of a methylenetetrahydrofolate-dehydrogenase 1958G/A polymorphism for neural tube defect risk J Hum Genet 2006 51 98 103 10.1007/s10038-005-0329-6 16315005

[B34] Strange RC Matharoo B Faulder GC Jones P Cotton W Elder JB Deakin M The human glutathione S-transferases: a case-control study of the incidence of the ST1 0 phenotype in patients with adenocarcinoma Carcinogenesis 1991 12 25 28 10.1093/carcin/12.1.25 1988177

[B35] Dhillon VS Shahid M Husain SA Associations of MTHFR DNMT3b 4977 bp deletion in mtDNA and GSTM1 deletion, and aberrant CpG island hypermethylation of GSTM1 in non-obstructive infertility in Indian men Mol Hum Reprod 2007 13 213 222 10.1093/molehr/gal118 17277043

[B36] Olteanu H Munson T Banerjee R Differences in the efficiency of reductive activation of methionine synthase and exogenous electron acceptors between the common polymorphic variants of human methionine synthase reductase Biochemistry 2002 41 13378 13385 10.1021/bi020536s 12416982

[B37] De Luca A Rizzardi M Buccino A Alessandroni R Salvioli GP Filograsso N Novelli G Dallapiccola B Association of dopamine D4 receptor (DRD4) exon III repeat polymorphism with temperament in 3-year-old infants Neurogenetics 2003 4 207 212 10.1007/s10048-003-0146-z 12687422

[B38] Wright AR Gatchel RJ Wildenstein L Riggs R Buschang P Ellis E Biopsychosocial differences between high-risk and low-risk patients with acute TMD-related pain J Am Dent Assoc 2004 135 474 483 15127871 10.14219/jada.archive.2004.0213

[B39] Herbig K Chiang EP Lee LR Hills J Shane B Stover PJ Cytoplasmic serine hydroxymethyltransferase mediates competition between folate-dependent deoxyribonucleotide and S-adenosylmethionine biosyntheses J Biol Chem 2002 277 38381 38389 10.1074/jbc.M205000200 12161434

[B40] Yamaza T Masuda KF Atsuta I Nishijima K Kido MA Tanaka T Oxidative stress-induced DNA damage in the synovial cells of the temporomandibular joint in the rat J Dent Res 2004 83 619 624 10.1177/154405910408300807 15271970

[B41] Sheets DW Jr Okamoto T Dijkgraaf LC Milam SB Schmitz JP Zardeneta G Free radical damage in facsimile synovium: correlation with adhesion formation in osteoarthritic TMJs J Prosthodont 2006 15 9 19 10.1111/j.1532-849X.2006.00063.x 16433646

[B42] Lee MC Kawai Y Shoji H Yoshino F Miyazaki H Kato H Suga M Kubota E Evidence of reactive oxygen species generation in synovial fluid from patient with temporomandibular disease by electron spin resonance spectroscopy Redox Rep 2004 9 331 336 10.1179/135100004225006830 15720828

[B43] Tomida M Ishimaru JI Murayama K Kajimoto T Kurachi M Era S Shibata T Intra-articular oxidative state correlated with the pathogenesis of disorders of the temporomandibular joint Br J Oral Maxillofac Surg 2004 42 405 409 10.1016/j.bjoms.2004.06.003 15336765

[B44] Milam SB Zardeneta G Schmitz JP Oxidative stress and degenerative temporomandibular joint disease: a proposed hypothesis J Oral Maxillofac Surg 1998 56 214 23 10.1016/S0278-2391(98)90872-2 9461148

